# Association of adverse childhood experiences with anemia in older Chinese: Guangzhou Biobank Cohort Study

**DOI:** 10.1038/s41598-024-54378-1

**Published:** 2024-02-27

**Authors:** Shao Yi He, Wei Sen Zhang, Chao Qiang Jiang, Ya Li Jin, Tai Hing Lam, Kar Keung Cheng, Lin Xu

**Affiliations:** 1 School of Public Health, Sun Yat-Sen University, No. 74, 2nd Zhongshan Road, Guangzhou, Guangdong China; 2 Guangzhou Twelfth People’s Hospital, Guangzhou, 510620 China; 3 School of Public Health, The University of Hong Kong, Hong Kong, China; 4 Institute of Applied Health Research, University of Birmingham, Birmingham, UK; 5 Greater Bay Area Public Health Research Collaboration, Guangzhou, China

**Keywords:** Medical research, Risk factors

## Abstract

To examine the association of adverse childhood experiences (ACEs) with anemia among older people. 24,116 participants aged 50 years or above were recruited. Multivariable linear and logistic regression was used to assess the associations of self-reported ACEs number with hemoglobin concentrations (g/dL) and presence of anemia. Older individuals with two or more ACEs, versus no ACEs, showed lower hemoglobin concentrations (β =  − 0.08 g/dL, 95% confidence intervals (CI) − 0.12 to − 0.03) and higher odds of anemia (odds ratio = 1.26, 95% CI 1.01–1.59). A more pronounced association between ACEs and anemia in the lower education group was found, while the association became non-significant in those with higher education (*P* for ACEs-education interaction = 0.02). ACEs was associated with anemia in older people, and the association was stronger in those with lower education, highlighting the significance of early-life psychological stressors assessment and consideration of education background in geriatric care.

## Introduction

Anemia is an important health and nutrition indicator. The Global Burden of Disease Study showed that the age-standardized prevalence of anemia was approximately 23% in 2019^1^. The prevalence in China was 15.2%, with a greater burden of disease in infants, fertile women and older people^2^. Anemia has been associated with higher risks of frailty and falls^3–5^, cognitive impairment^4^ and all-cause mortality^6^ in older people. Identifying risk factors of anemia can facilitate preventive measures in individuals and help public health officials develop targeted interventions and programs to address the underlying causes and reduce the burden of the condition on healthcare systems and society as a whole.

Adverse childhood experiences (ACEs) including abuse, neglect and household dysfunction have been linked to long-term adverse health effects throughout one’s life-course^7^, such as diabetes^8^, renal function decline^9^ and cardiovascular disease (CVD)^10^. As a nonspecific, comorbid manifestation of these chronic diseases^11–13^, it is hypothesized that ACEs may also lead to anemia in older people. Furthermore, ACEs was associated with higher levels of adulthood psychological stress^14,15^, and the latter could subsequently lead to anemia^16^. Notably, a study showed that children with domestic violence were more likely to be anemic, suggesting that childhood psychological stress might play a role in the pathogenesis of anemia^17^. However, whether ACEs have any impact on anemia during later life is largely unclear. Therefore, this study examined the association between ACEs and anemia in older Chinese using baseline data from a well-established population-based cohort study.

## Results

**Figure 1:**
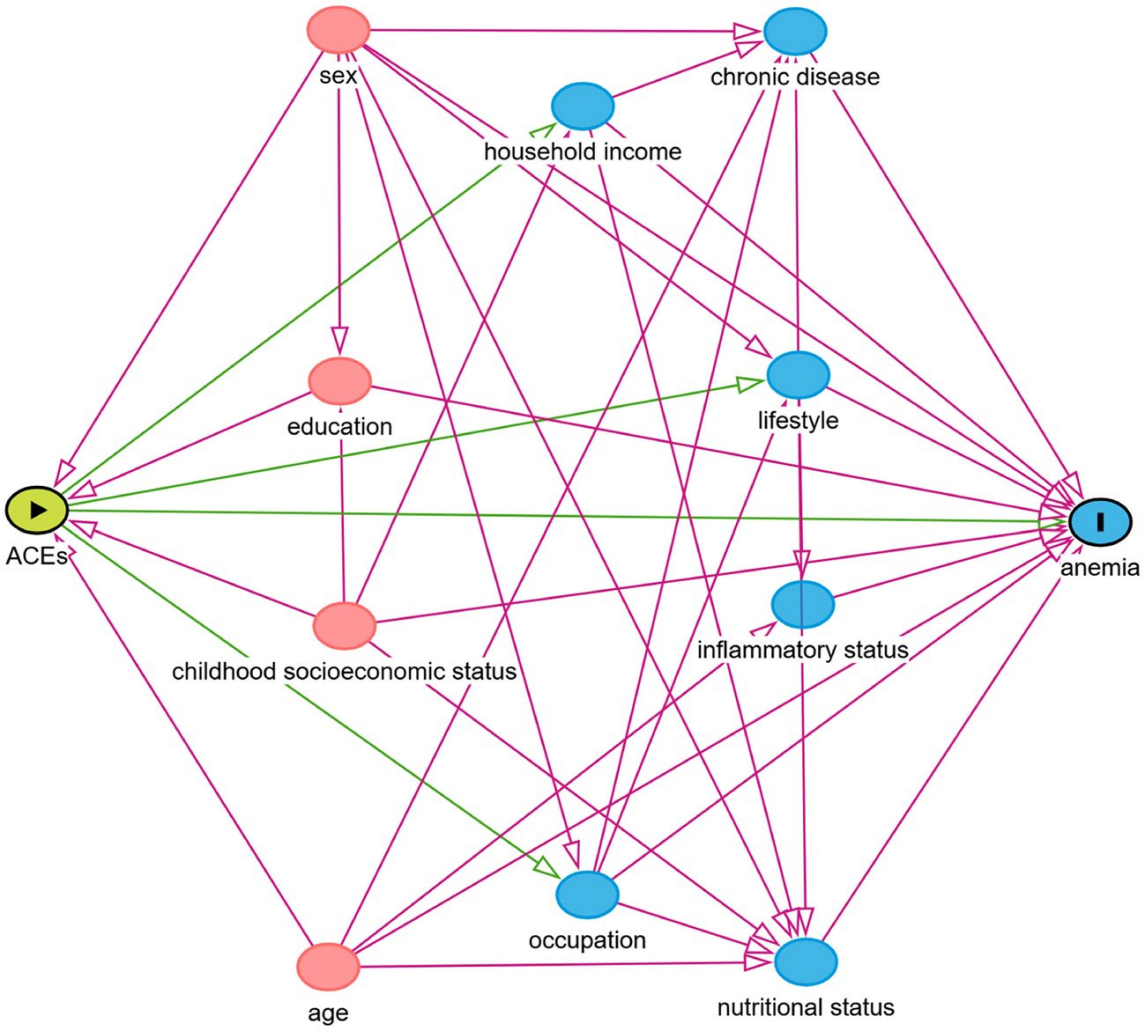
Directed acyclic graph (DAG) of factors associated with anemia in Guangzhou Biobank Cohort Study. *ACEs* adverse childhood experiences; *BMI* body mass index. *Note* Green lines indicated causal paths. Pink lines indicated biasing paths. Pink dots were potential confounders which were ancestors of exposure and outcome. Blue dots were latent mediators.

Of 30,430 GBCS participants, after excluding those with missing data on ACEs and hemoglobin (N = 6314), 24,116 participants were included in the present study. The mean age was 60.7 (standard deviation (SD) = 7.1) years. Of them, 12,825 (53.2%) experienced at least one ACEs and 4843 (20.1%) experienced two or more ACEs. The prevalence of anemia was 2.7%. Figure 1 illustrates the causal pathways between ACEs and anemia. The pink dots, encompassing sex, age, CSES and education, are identified as primary confounders, and the blue dots, including occupation, lifestyle factors, household income and BMI are considered potential mediators (Fig. 1).

Participants who were men, aged 60 years or above, with lower education, lower household income and CSES had more ACEs (all *P* < 0.001) (Table 1). In addition, participants who had more ACEs had higher proportion of current smokers and alcohol users, were more physically inactive, and had higher prevalence of anemia (*P* from < 0.001 to 0.02) (Table 1). However, no significant difference in BMI levels by the ACE number was found (*P* = 0.35) (Table 1).

**Table 1:** Characteristics of participants by number of ACEs in the Guangzhou Biobank Cohort Study.

	Number of ACEs			
	0	1	≥ 2	*P*^a^
N	11,291	7982	4843	
Sex, %				
Women	76.6	71.1	66.4	< 0.001
Men	23.4	28.9	33.6	
Age group, %, y				
≤ 59	56.4	47.2	34.7	< 0.001
60–69	34.3	38.6	43.9	
≥ 70	9.3	14.2	21.4	
Occupation, %				
Manual	61.5	58.1	59.3	< 0.001
Non-manual	21.9	24.5	24.0	
Other	16.6	17.4	16.7	
Education status, %				
Primary or below	37.4	39.1	46.6	< 0.001
Middle school	54.0	51.5	43.5	
College or above	8.6	9.5	9.9	
Household annual income, %, CNY				
< 30,000	36.3	36.7	39.7	< 0.001
≥ 30,000	42.5	41.1	39.0	
Don’t known	21.2	22.3	21.6	
Childhood socioeconomic status score, mean (SD)	4.0 (1.7)	4.3 (1.6)	4.6 (1.4)	< 0.001
Body mass index, mean (SD), kg/m^2^	23.8 (3.3)	23.8 (3.3)	23.7 (3.2)	0.32
Smoke status, %				
Never	84.3	80.4	77.6	< 0.001
Ever	15.8	19.6	22.4	
Alcohol use, %				
Never	65.0	64.5	62.3	0.002
Former user	2.3	2.6	3.2	
Current user	32.7	32.9	34.6	
Physical activity, %				
Inactive	9.1	9.9	9.4	0.002
Moderate	40.1	41.6	42.6	
Active	50.8	48.5	48.0	
Hgb, mean (SD), g/dL	13.6 (1.3)	13.7 (1.4)	13.7 (1.4)	0.04
Anemia, %				
No	97.6	97.2	96.9	0.02
Yes	2.4	2.8	3.1	

*ACEs* adverse childhood experiences; *Hgb* hemoglobin; *SD* standard deviation.

^a^*P* value in analysis of variance for continuous variables, and in chi-square test for categorical variables.

The prevalence of anemia was highest in participants with two or more ACEs (3.1%) followed by those with one ACEs (2.8%) and without ACEs (2.4%) (Table 1), and a dose–response relationship was found in the association of ACEs with anemia and hemoglobin concentrations (*P* for trend = 0.030 and 0.001, respectively) (Table 2). After adjusting for sex, age, education and CSES, compared to those without ACEs, participants with two or more ACEs had a 26% higher risk for prevalence of anemia (OR = 1.26, 95% CI 1.01–1.59) than those without ACEs (Table 2). Similar association was also found in Model 2 and Model 3 after adjusting for potential mediators (OR = 1.27, 95% CI 1.01–1.59 and 1.33, 95% CI 1.04–1.69, respectively) (Table 2). Participants with two or more ACEs showed lower hemoglobin concentrations (*β* =  − 0.08 g/dL, 95% CI − 0.12 to − 0.03) and result remained in Model 2 and Model 3 (*β* =  − 0.08 g/dL, 95% CI − 0.12 to − 0.04 and − 0.06 g/dL, 95% CI − 0.11 to − 0.02, respectively) (Table 2). After stratifying by sex, the associations of greater number of ACEs with lower hemoglobin concentrations remained (adjusted β and 95% CI − 0.12 (− 0.21 to − 0.03) in men and − 0.16 (− 0.12 to − 0.01) in women) (Table 2). The adjusted OR for anemia became non-significant in both sexes probably due to insufficient sample size (adjusted ORs and 95% CI 1.28 (0.83–1.96) in men and 1.26 (0.96–1.65) in women) (Table 2).

**Table 2:** Association of number of ACEs with hemoglobin concentrations (g/dL) and anemia by sex in Guangzhou Biobank Cohort Study.

Number of ACEs	Number of participants	Model 1^a^	Model 2^b^	Model 3^c^
Total (N = 24,116)				
		Adjusted *OR* (95% *CI*) of anemia		
0	11,291	Reference (1)	Reference (1)	Reference (1)
1	7982	1.21 (0.99–1.48)	1.22 (1.00–1.50)	1.24 (1.00–1.55)*
≥ 2	4843	1.26 (1.01–1.59)*	1.27 (1.01–1.59)*	1.33 (1.04–1.69)*
*P* for trend		0.03	0.03	0.02
		Adjusted *β* (95% *CI*) of Hgb concentrations (g/dL)		
0	11,291	Reference (0)	Reference (0)	Reference (0)
1	7982	− 0.01 (− 0.05 to 0.02)	− 0.02 (− 0.05 to 0.02)	− 0.02 (− 0.06 to 0.02)
≥ 2	4843	− 0.08 (− 0.12 to − 0.03)**	− 0.08 (− 0.12 to − 0.04)***	− 0.06 (− 0.11 to − 0.02)**
*P* for trend		0.001	0.001	0.01
Men (N = 6577)				
		Adjusted *OR* (95% *CI*) of anemia		
0	2645	Reference (1)	Reference (1)	Reference (1)
1	2305	1.24 (0.83–1.85)	1.24 (0.83–1.85)	1.06 (0.69–1.63)
≥ 2	1627	1.28 (0.83–1.96)	1.22 (0.79–1.88)	1.15 (0.73–1.82)
*P* for trend		0.25	0.35	0.54
		Adjusted *β* (95% *CI*) of Hgb concentrations (g/dL)		
0	2645	Reference (0)	Reference (0)	Reference (0)
1	2305	− 0.02 (− 0.10 to 0.06)	− 0.02 (− 0.10 to 0.06)	− 0.01 (− 0.10 to 0.07)
≥ 2	1627	− 0.12 (− 0.21 to − 0.03)*	− 0.12 (− 0.21 to − 0.03)*	− 0.11 (− 0.21 to − 0.02)*
*P* for trend		0.02	0.02	0.02
Women (N = 17,539)				
		Adjusted *OR* (95% *CI*) of anemia		
0	8646	Reference (1)	Reference (1)	Reference (1)
1	5677	1.20 (0.95–1.52)	1.21 (0.96–1.53)	1.31 (1.02–1.69)*
≥ 2	3216	1.26 (0.96–1.65)	1.28 (0.98–1.69)	1.40 (1.05–1.87)*
*P* for trend		0.07	0.05	0.01
		Adjusted *β* (95% *CI*) of Hgb concentrations (g/dL)		
0	8646	Reference (0)	Reference (0)	Reference (0)
1	5677	− 0.01 (− 0.05 to 0.03)	− 0.01 (− 0.06 to 0.03)	− 0.01 (− 0.05 to 0.03)
≥ 2	3216	− 0.06 (− 0.12 to − 0.01)*	− 0.06 (− 0.11 to − 0.01)*	− 0.04 (− 0.09 to 0.01)
*P* for trend		0.03	0.02	0.17

*ACEs* adverse childhood experiences; *Hgb* hemoglobin; *OR* odds ratio; *CI* confidence interval.

^a^Model 1 adjusted for sex, age, education status and childhood socioeconomic status.

^b^Model 2 additionally adjusted for occupation and household annual income.

^c^Model 3 additionally adjusted for lifestyle factors (smoke status, alcohol use and physical activities), body mass index, white blood count and chronic disease status.

*P < 0.05; **P < 0.01; ***P < 0.001.

**Figure 2:**
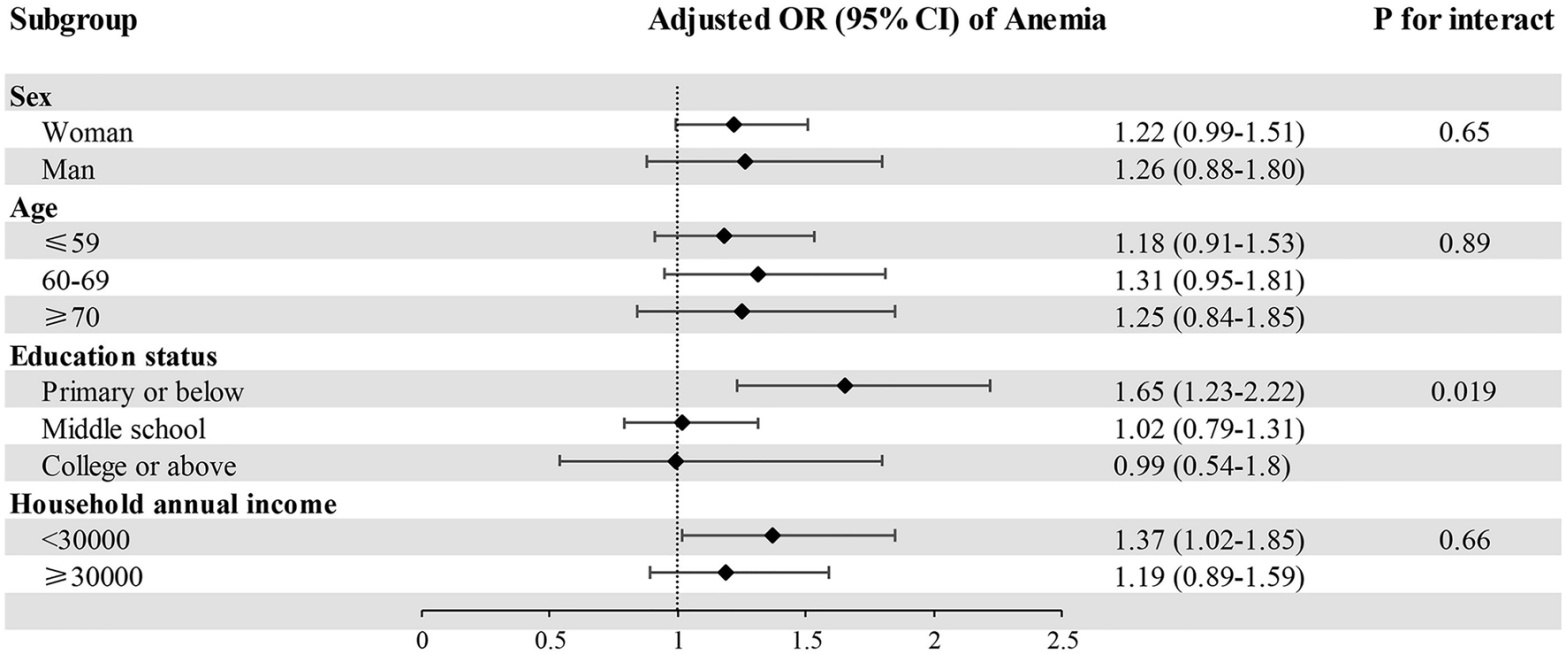
Association of ACEs (Yes vs. No) with anemia by demographic characteristics and socioeconomic status in Guangzhou Biobank Cohort Study. *OR* odds ratio; *CI* confidence interval, *ACEs* adverse childhood experiences. *Note* This model adjusted for sex, age, education status and childhood socioeconomic status.

Figure 2 shows a significant interaction between ACEs and education (*P* for interact = 0.02) on anemia, but no interaction with sex, age and household annual income (*P* for interact from 0.65 to 0.89). In participants with education of primary school or below, those with at least one ACEs, versus without ACEs, had 65% higher odds for anemia (OR = 1.65, 95% CI 1.23–2.22).

Results of sensitive analysis reported the association between ACEs and hemoglobin concentrations by education. Although stratification analysis showed no significant interaction between ACEs and education on hemoglobin concentrations (*P* for interact = 0.32), compared to those with higher educational level, more ACEs were associated with a greater decrease of hemoglobin in those with poorer education (Supplementary Table S1). Table 3 shows that, compared to participants without ACEs, those with two or more ACEs also had a significantly negative association with hematocrit value (total participants *β* =  − 0.25%, 95% CI − 0.39 to − 0.11; men *β* =  − 0.37%, 95% CI − 0.62 to − 0.12 and women *β* =  − 0.18%, 95% CI − 0.35 to − 0.02), with a dose–response pattern (*P* for trend from 0.001 to 0.02). Participants experiencing two or more ACEs had significantly higher odds for the lowest hemoglobin quintile, while the association was non-significant in men. Moreover, we found a non-significantly positive association between ACEs and anemia defined by the WHO criteria (Table 3). After excluding 2195 participants with hypochromic anemia, significant associations between more ACEs and lower hemoglobin concentrations remained (Supplementary Table S2). In Supplementary Table S3, we further examined the associations of specific ACEs with anemia defined by WHO criteria and hemoglobin concentrations. We found that in five dimensions, only separation from mother was significantly associated with higher odds of anemia and lower hemoglobin concentrations in all participants. Similar results were found in both men and women.

**Table 3:** Association of number of ACEs with different measurements of anemia by sex in Guangzhou Biobank Cohort Study.

Number of ACEs	Total ^a^	Men ^a^	Women ^a^
	Adjusted *β* (95% *CI*) of HCT (%)		
0	Reference (0)	Reference (0)	Reference (0)
1	− 0.10 (− 0.22 to 0.02)	− 0.04 (− 0.26 to 0.18)	− 0.13 (− 0.27 to 0.01)
≥ 2	− 0.25 (− 0.39 to − 0.11)***	− 0.37 (− 0.62 to − 0.12)**	− 0.18 (− 0.35 to − 0.02)*
*P* for trend	0.001	0.006	0.02
	Adjusted *OR* (95% *CI*) for the lowest hemoglobin quintile^†^		
0	Reference (1)	Reference (1)	Reference (1)
1	1.00 (0.93–1.09)	0.99 (0.85–1.16)	1.01 (0.92–1.10)
≥ 2	1.12 (1.02–1.22)*	1.12 (0.94–1.32)	1.11 (1.00–1.24)
*P* for trend	0.03	0.24	0.08
	Adjusted *OR* (95% *CI*) of anemia defined by WHO^‡^		
0	Reference (1)	Reference (1)	Reference (1)
1	1.01 (0.91–1.12)	0.95 (0.76–1.19)	1.02 (0.91–1.15)
≥ 2	1.08 (0.96–1.21)	1.03 (0.81–1.31)	1.09 (0.95–1.25)
*P* for trend	0.26	0.84	0.26

*ACEs* adverse childhood experiences; *HCT* hematocrit value; *OR* odds ratio; *CI* confidence interval.

^a^Adjusted for sex, age, education status and childhood socioeconomic status.

^**†**^Lowest (1^st^) hemoglobin quintile (sex-specific) *versus* quintiles 2^nd^ to 5^th^.

^**‡**^Anemia defined by WHO: hemoglobin concentrations < 13.0 g/dL for men and 12.0 g/dL for women.

*P < 0.05; **P < 0.01; ***P < 0.001.

## Discussion

To the best of our knowledge, this is the first study showing a positive dose–response association between ACEs and anemia in older people, taking into account childhood demographic and socioeconomic characteristics. Specifically, the greater number of ACEs, the higher odds of anemia and lower levels of hemoglobin in older adulthood. However, this association appeared to be modified by educational attainment, with the association being less pronounced in those with higher education. The association of ACEs with anemia appeared to be non-significant in men. Regarding specific ACEs, the separation appeared as the most robust predictor for anemia. Our results demonstrated the potential influence of early-life determinants on anemia in later life, and highlighted the importance of educational preventive interventions throughout the life-course.

Our findings were consistent with previous studies showing that men, older individuals, and those with lower levels of education and income were more likely to have experienced adversity during their childhood^18–20^. Moreover, individuals with an increased number of ACEs exhibited a greater propensity to adopt unhealthy lifestyles, such as smoking, alcohol consumption, and physical inactivity, as evidenced by previous research^21–23^. ACEs assessed in our study tended to reflect early-life psychological stressor. In addition, our study for the first time investigated the long-term association between accumulated early psychological stress and anemia in the elderly. Previous studies are relatively limited and lacking of direct evidence^17,24^. A cross-sectional study reported a positive association between maternal reported domestic violence and anemia of Indian children using unadjusted model^17^. Another recent systematic review including 18 studies found that a history of girl child marriage was significantly and positively associated with anemia (low hemoglobin) in adolescence^24^. The two studies above indicated a possible correlation of childhood psychological stress patterned by domestic and gender-based violence with anemia. However, both the two studies only used single adverse event as exposures and early anemia as outcomes, and the latter (childhood anemia) is closely associated with low childhood household wealth, especially in low- and middle-income countries^25^. Among older generation in China, individuals with more ACEs generally were more likely to have grown up in harsh conditions marked by material deprivation, resulting in poorer childhood nutrition^26^. Several previous studies also reported a positive association of nutritional early life adversity (i.e., foster experience or famine) with anemia^27,28^. Among these studies, only one explored the association between exposure to the Chinese famine during early life and the risk of anemia in adulthood^28^. The study found that individuals who were exposed to famine in utero had a higher risk of anemia in later life. However, it could not rule out the possibility of a similar association in individuals exposed to famine postnatally, given that the famine persisted for three years rather than only during the pregnancy period^28^. Therefore, main results of the present study were adjusted for CSES to avoid its potential confounding effect on the association of ACEs with anemia. Our finding suggests that the relationship between ACEs and anemia in the elderly is still significant after excluding the potential influence of childhood nutrition.

The findings from our subgroup analyses suggest that the association between ACEs and anemia in older people was consistent across age, sex, and income strata. However, those with lower education had higher susceptibility to anemia attributable to ACEs. There were two possible explanations regarding the interaction between ACEs and education status. One explanation postulate that individuals with higher education status tend to prioritize their health more proactively by adopting healthier lifestyles and taking positive measures to prevent or treat diseases, which may alleviate the adverse effect of ACEs. Another plausible explanation suggests that a good education typically reflects a more favorable childhood living environment, which can have long-term benefits for both physical and mental health. For instance, a previous study showed that the elderly who had attended the University of the Third Age (U3A) for six months or longer had greater psychological adjustment and a higher level of satisfaction to life compared with new entrants, indicating the positive effects of continuing education^29^. Further studies are needed to clarify the distinct influences and interactions that early and late education may have on their quality of life. Furthermore, there exists a possibility to investigate whether late-life education can mitigate or counteract long-term adverse health effects due to ACEs.

Existing evidence from animal-based experiments and population-based studies generally support the association of ACEs with anemia in the elderly. ACEs were associated with greater psychological stress^30,31^, and the latter can increase the risk of oxidative stress^32,33^. In addition, higher oxidative stress may damage red blood cells and subsequently lead to hemolytic anemia^34,35^. While acute stress has been linked with immune-boosting properties and overall biological fitness over short periods^36,37^, chronic stress has been associated with accelerated biological aging and dysregulation of the hypothalamic–pituitary–adrenal (HPA) axis homeostasis^38^. Two animal experiments have shown positive association between chronic stress and iron deficiency anemia (IDA)^39,40^. Prolonged chronic stress and persistent iron dysregulation were found to prevent anemia recovery following trauma^39^, whereas the removal of chronic stress after trauma improved erythroid progenitor growth and hemoglobin concentrations^40^. Moreover, another experimental study suggested that pure psychological stress reduced serum iron and inhibited erythropoiesis in rats^41^. Psychological stress was also associated with lower iron levels and iron deficiency at 12 months in infants exposed to early life adversity^42^. One possible inference is that ACEs could result in chronic activation of the stress response system, leading to dysfunction of the autonomic, neuroendocrine, and inflammatory systems and, consequently, unfavorable outcomes later in life^10^. However, we failed to verify the possibly biological mechanism with missing information on related indicators. Besides, our results adjusting for other potential mediators in model 2 and 3 showed that the association between ACEs and higher risk of anemia could not be fully explained by demographic characteristics and lifestyle factors. Further studies investigating other potential mediators, such as inflammation and nutrition status throughout the life-course, are warranted.

This study exhibited a significant strength by determining reasonable potential confounding variables via DAG. Several recent studies that explored the association of ACEs with health outcome in adulthood, disregarded the possibility of "overadjustment bias"^43^ when constructing adjustment models. It is important to note that adulthood exposures which might have impact on outcomes cannot affect exposures before the age of 18 years. Further strengths of this study encompassed a sufficient sample size and the accurate measurement of the hemoglobin concentrations^44^. Our study also had several limitations. First, recall error could not be avoided because the information of ACEs was collected by self-report, although the recall error tended to be non-differential. Moreover, as older Chinese were generally less perceptive to ACEs due to the cultural specificity^45^, the prevalence of ACEs could be underestimated. In addition, compared to the specific instruments used in other studies, some well-established ACEs indicators were not included in our study. Second, as our study samples were from a developed city in China, the prevalence of anemia was low. But we additionally used other definitions of anemia^6^ to explore the dose–response association and showed similar results. Third, although our sensitivity analysis excluding participants at high risk of thalassemia showed similar results, the diagnosis of thalassemia was based on hematological indicators rather than physician diagnosis. Therefore, other types of acquired anemia, such as IDA, might be excluded inappropriately. Finally, we were unable to test the external validity of our CSES questionnaire, as it was not assessed against other established instruments. While we endeavored to ensure the internal consistency and relevance of our questionnaire to our study population, future research would benefit from comparing these findings with those obtained using other validated instruments, to further confirm the generalizability of our results.

In conclusion, our study showed that the presence of two or more ACEs was associated with lower hemoglobin concentrations and a 26% increase in the odds of anemia in older people. The association was more pronounced in older people with lower education, suggesting that education attainment may mitigating the adverse effect of ACEs on anemia.

## Methods

### Study sample

Guangzhou Biobanking Cohort Study (GBCS) is a 3-way collaboration of the Guangzhou Twelfth People's Hospital and the Universities of Hong Kong and Birmingham. Details of the GBCS have been described previously^44^. From 2003 to 2008, 30,430 participants aged 50 years or above were recruited from the Guangzhou Health and Happiness Association for the Respectable Elders (GHHARE), a large unofficial welfare organization with branches in all ten districts of Guangzhou. Membership of GHHARE was eligible to permanent residents of Guangzhou aged 50 years or above, approximately 95,000, accounting for 7% of the total elderly population in Guangzhou, and available for a nominal fee of RMB four Yuan (about 50 US cents) per month. Participants were recruited in three phases, and selected from the membership of GHHARE. Participants were included if they are permanent Guangzhou residents capable of consenting and have agreed to participate, ambulatory, and not receiving treatment modalities, which, if omitted, may result in immediate life-threatening risk such as chemotherapy or radiotherapy for cancer, or dialysis for renal failure. Participants who did not consent, were mentally or cognitively abnormal, unable to care for themselves, or had mobility difficulties were excluded^44^. After excluding 6314 participants lacking information of interest (ACEs exposure and hemoglobin concentrations), 24,116 participants were used included in the current study. Information of demographic characteristics, lifestyle factors, and personal and family disease history was collected by face-to-face interview using a computer-assisted questionnaire by trained nurses. Physical examinations were conducted by full-time physicians. The GBCS was approved by the Guangzhou Medical Ethics Committee of the Chinese Medical Association. All participants provided written informed consent before participation. We confirm that all experiments were performed in accordance with relevant guidelines and regulations.

### Exposures

In GBCS dataset, five items are used to measure the number of ACEs. These five items reflected separation, traumatic experience, emotional abuse, domestic violence and parental death respectively, which have been considered in previous ACEs studies, such as the National Population Health Survey (NPHS) of the Canadian population and the China Health and Retirement Longitudinal Study (CHARLS)^46–48^. These questions were more suitable for older Chinese and the unique cultural background they grew up in. GBCS defined each items using dichotomous variable (Yes/No), and cumulate the self-reported ACEs number to calculate a total score analogous to previous questionnaires^49,50^. Detail of categories of each item is shown in Supplementary Table S4. Furthermore, we categorized the participants into three groups according to the total score of each individual (0, 1 and ≥ 2 ACEs).

### Outcome

Study outcomes included plasma hemoglobin concentrations (g/dL, measured by Sysmex KX21 Hematology Analyzer, Japan) and presence of anemia^44^. Anemia was defined by hemoglobin concentrations of less than 12.0 g/dL for men and less than 11.0 g/dL for women^51^.

### Covariates

Covariates included demographic characteristics, behavioral lifestyle, and childhood socioeconomic status. Demographic characteristics included age, sex, education, household income and occupation. Behavioral lifestyle included smoking, alcohol consumption and physical activity. Information of smoking status (never/ever) and alcohol use (never/former/current user) were collected by a standardized questionnaire. Smoking was defined as having smoked at least one cigarette per day or seven cigarettes per week for at least half a year. Physical activity was assessed using a validated Chinese version of the International Physical Activity Questionnaire (IPAQ), and categorized as inactive, moderate and active level^52^. Childhood socioeconomic status (CSES) was evaluated using questions accounting for the cultural and historical contexts of mid-20th-century southern China, including an index of parental material possessions during one’s childhood (bike, sewing, watch), childhood hunger, childhood meat eating frequency and purchased new clothes on special occasions. The questions are all derived from the dimension representing childhood material deprivation, with Cronbach's alpha of 0.74. The content of questionnaire and score of each item are shown in Supplementary Table S4. Total score ranges from zero to six, and a higher score indicates lower CSES. In addition, plasma white blood count (WBC) was measured as an indicator of inflammation by Sysmex KX21 Hematology Analyzer. Body mass index (BMI) is commonly used to assess the nutritional status. We also defined poor self-reported health status by any of the following: (1) regular use of medications for chronic diseases, such as diabetes, hypercholesterolemia, or cardiovascular diseases, (2) any hospital admission in the past 6 months before baseline examination, (3) self-reported cardiovascular disease history, or (4) self-reported cancer history^53^. These conditions are regarded as presence of chronic disease status.

### Statistical analysis

Characteristics of participants were reported as mean (SD) for continuous variables and frequency for categorical variables by the numbers of ACEs. Chi-square test and one-way analysis of variance (ANOVA) were used to compare categorical and continuous variables respectively. The directed acyclic graph (DAG) was employed for initial identification of conditional dependencies and causal relationships between variables, as well as for the demonstration of the primary confounders requiring adjustment^54^.

Moreover, we used linear regression and logistic regression to assess the association of number of ACEs with hemoglobin concentrations and anemia, respectively. Three models were developed with adjustment for different covariates. Model 1 was adjusted for sex, age, education, and childhood socioeconomic status based on DAGs. This was deemed appropriate since a distinct temporal sequence existed between ACEs and anemia in the elderly, and events that occurred beyond the age of 18 were not considered confounders of the association between ACEs and anemia in the elderly. To explore potential mediating effects, Model 2 was additionally adjusted for other demographic factors and Model 3 was further adjusted for lifestyle factors, including smoking status, alcohol use, physical activity. Body mass index, chronic diseases, as well as inflammatory status were also adjusted in Model 3. Furthermore, we also conducted interaction tests and stratification analysis to explore potential moderation effect of sex, age, education or income level, and analyzed in men and women separately. As hematocrit (%) is another indicator of anemia^55^, sensitivity analyses on the associations between ACEs and hematocrit were done. Moreover, another sensitivity analysis excluding participants with MCV less than 78 femtoliter (fL) or MCH less than 25.5 picogram (pg), who were considered to have hypochromic anemia and thalassemia, was perform^56,57^. We also tested the associations of each specific ACEs with anemia. Stata 16.0 version (STATA Corp LP, College Station, Texas, USA) was used for statistical analysis. All tests were two-sided and *P* < 0.05 was statistically significant.

### Supplementary Information

Supplementary Tables.

## Data Availability

All data generated or analyzed during this study are included in this article. Further enquiries can be directed to the corresponding author.
