# Supplementary material for: Association of adverse childhood experiences with anemia in older Chinese: Guangzhou Biobank Cohort Study
Source: Sci Rep. 2024 Feb 27;14:4729. doi: 10.1038/s41598-024-54378-1 (PMC10899217; doi:10.1038/s41598-024-54378-1)
Supplement: Supplementary file 1 — Supplementary Tables. [file 41598_2024_54378_MOESM1_ESM.pdf]

Association of adverse childhood experiences with anemia in older Chinese: Guangzhou Biobank Cohort Study

Shao Yi He, MPhil candidate. (heshy55@mail2.sysu.edu.cn) [a e], Wei Sen Zhang, Ph.D. (zwscn@hotmail.com) [b e#], Chao Qiang Jiang, M.D. (cqjiang@hku.hk) [b e], Ya Li Jin, MPH (jinyali22@163.com) [b e], Tai Hing Lam, Ph.D. (hrmrlth@hku.hk) [c e], Kar Keung Cheng, Ph.D. (K.K.Cheng@bham.ac.uk) [d e], Lin Xu, Ph.D. (xulin27@mail.sysu.edu.cn) [a c d e#]

[a] School of Public Health, Sun Yat-sen University, Guangzhou, China

[b] Guangzhou Twelfth People's Hospital, Guangzhou, China

[c] School of Public Health, The University of Hong Kong, Hong Kong, China

[d] Institute of Applied Health Research, University of Birmingham, Birmingham, UK

[e] Greater Bay Area Public Health Research Collaboration

[#] Co-corresponding author

Corresponding to:

Corresponding author (1): Professor WS Zhang

Guangzhou Twelfth People’s Hospital, Guangzhou 510620, China

Email: zwsgzcn@163.com

Corresponding author (2): Professor Lin Xu

School of Public Health, Sun Yat-sen University

No. 74, 2nd Zhongshan Road, Guangzhou, Guangdong, China

Telephone: 020-87335523

E-mail: xulin27@mail.sysu.edu.cn

Supplementary material

Supplementary Table S1. Association of number of ACEs with hemoglobin concentrations (g/dL) by education status in Guangzhou Biobank Cohort Study

Number of ACEs	No.	Adjusted *β* (95% *CI*) of concentrations of HGB (g/dL)		
		Model 1[a]	Model 2[b]	Model 3[c]
Primary or below (N=9596)				
0	4223	Reference (0)	Reference (0)	Reference (0)
1	3117	-0.04 (-0.10 to 0.02)	-0.04 (-0.10 to 0.02)	-0.05 (-0.11 to 0.02)
≥2	2256	-0.10 (-0.17 to -0.04)**	-0.11 (-0.17 to -0.04)**	-0.09 (-0.15 to -0.02)*
P for trend		0.002	0.002	0.013
Middle school or above (N=14,511)				
0	7065	Reference (0)	Reference (0)	Reference (0)
1	4862	0.00 (-0.04 to 0.05)	-0.00 (-0.05 to 0.05)	0.00 (-0.05 to 0.05)
≥2	2584	-0.06 (-0.11 to 0.01)	-0.06 (-0.12 to -0.01)*	-0.06 (-0.12 to -0.01)*
P for trend		0.11	0.076	0.084
P for interact		0.32		

Abbreviations: ACEs, adverse childhood experiences; Hgb, hemoglobin; CI, confidence interval.

[a] Model 1 adjusted for sex, age and childhood socioeconomic status.

[b] Model 2 additionally adjusted for occupation and household annual income.

[c] Model 3 additionally adjusted for body mass index and lifestyle (smoke status, alcohol use and physical activities).

*: $P<0.05$; **: $P<0.01$; ***: $P<0.001$

Supplementary Table S2. Association of number of ACEs with hemoglobin concentrations (g/dL) excluding participants with hypochromic anemia in Guangzhou Biobank Cohort Study (N=21,921)

Number of ACEs	No.	Adjusted β (95% *CI*) of Hgb concentrations (g/dL)		
		Model 1[a]	Model 2[b]	Model 3[c]
0	10,263	Reference (0)	Reference (0)	Reference (0)
1	7270	-0.02 (-0.06 to 0.01)	-0.02 (-0.06 to 0.01)	-0.02 (-0.06 to 0.01)
≥2	4388	-0.08 (-0.12 to -0.04)***	-0.08 (-0.12 to -0.04)***	-0.07 (-0.11 to -0.03)**
P for trend		<0.001	<0.001	0.001

Abbreviations: ACEs, adverse childhood experiences; Hgb, hemoglobin; CI, confidence interval.

[a] Model 1 adjusted for sex, age, education status and childhood socioeconomic status.

[b] Model 2 additionally adjusted for occupation and household annual income.

[c] Model 3 additionally adjusted for body mass index and lifestyle (smoke status, alcohol use and physical activities).

*: $P<0.05$; **: $P<0.01$; ***: $P<0.001$

Supplementary Table S3. Association of specific ACEs with anemia and hemoglobin concentrations by sex in Guangzhou Biobank Cohort Study.

ACEs categories (Yes *vs.* No)	Number (%) of total [a]	Total	Men	Women
	Adjusted *OR* (95% *CI*) of anemia [b] (Ref: No) [c]			
Separation	7729 (32.1%)	1.13 (1.02-1.24)*	1.08 (0.89-1.31)	1.14 (1.02-1.27)*
Traumatic experience	2563 (10.6%)	1.00 (0.87-1.16)	1.06 (0.79-1.42)	0.98 (0.83-1.16)
Emotional abuse	572 (2.4%)	1.03 (0.77-1.39)	1.34 (0.85-2.12)	0.89 (0.60-1.31)
Domestic violence	1812 (7.5%)	0.87 (0.73-1.03)	0.89 (0.61-1.29)	0.86 (0.70-1.05)
Parental death	6013 (24.9%)	1.03 (0.93-1.14)	0.99 (0.81-1.21)	1.04 (0.92-1.17)
	Adjusted β (95% *CI*) of hemoglobin concentrations (Ref: No) [c]			
Separation	7729 (32.1%)	-0.08 (-0.12 to -0.05)***	-0.09 (-0.17 to -0.02)*	-0.08 (-0.11 to -0.04)***
Traumatic experience	2563 (10.6%)	-0.04 (-0.10 to 0.01)	-0.08 (-0.19 to 0.04)	-0.03 (-0.09 to 0.03)
Emotional abuse	572 (2.4%)	-0.06 (-0.16 to 0.05)	-0.07 (-0.25 to 0.12)	-0.06 (-0.18 to 0.07)
Domestic violence	1812 (7.5%)	0.01 (-0.05 to 0.07)	-0.05 (-0.19 to 0.08)	0.03 (-0.03 to 0.10)
Parental death	6013 (24.9%)	-0.02 (-0.05 to 0.02)	-0.05 (-0.13 to 0.02)	0.00 (-0.04 to 0.04)

Abbreviations: ACEs, adverse childhood experiences; *vs.*, versus; OR, odds ratio; CI, confidence interval.

[a] Number: the number (proportion) of participants that self-reported with each specific ACE;

[b] Anemia defined by WHO: hemoglobin concentrations < 13.0 g/dL for men and 12.0 g/dL for women.

[c] Adjusted for sex, age, education status and childhood socioeconomic status.

*: $P<0.05$; **: $P<0.01$; ***: $P<0.001$

Supplementary Table S4. Categories and Items of adverse childhood experiences questionnaire in GBCS

**Category**	**Items of the questionnaire**	**Score**
	(Prior to 18 years old, did you experience any of the following?)	
Parental death	Early death of any parents.	Yes=1; No=0
Separation	Separation from mother for more than one year continuously.	Yes=1; No=0
Traumatic experience	An experience so frightening as to be thought about for years afterwards.	Yes=1; No=0
Emotional abuse	Being sent away from home because of doing something wrong.	Yes=1; No=0
Domestic violence	Your parents arguing and fighting frequently.	Yes=1; No=0

Notes: The total score ranges from zero to five.

Supplementary Table S5. Categories and Items of childhood socioeconomic status questionnaire in GBCS

**Items of the questionnaire**	**Score**
Did your parents own a bicycle when you were a child?	Yes=1; No=0
Did your parents own a sewing machine when you were a child?	Yes=1; No=0
Did your parents own a watch when you were a child?	Yes=1; No=0
Did you usually get new clothes at Chinese New Year?	Yes=1; No=0
How often do you remember being hungry as a child?	Ever=1; Never=0
How often did you eat meat as a child?	Not daily=1; Daily=0

Notes: The total score ranges from zero to six.
